# Oxidation of the cysteine-rich regions of parkin perturbs its E3 ligase activity and contributes to protein aggregation

**DOI:** 10.1186/1750-1326-6-34

**Published:** 2011-05-19

**Authors:** Fanjun Meng, Dongdong Yao, Yang Shi, Jonathan Kabakoff, Wei Wu, Joshua Reicher, Yuliang Ma, Bernd Moosmann, Eliezer Masliah, Stuart A Lipton, Zezong Gu

**Affiliations:** 1 Department of Pathology & Anatomical Sciences, Center for Translational Neuroscience, University of Missouri-Columbia School of Medicine, Columbia, MO, USA; 2 Del E. Webb Center for Neuroscience, Aging, and Stem Cell Research, Sanford-Burnham Medical Research Institute, La Jolla, CA, USA; 3 Beijing Institute of Genomics, Chinese Academy of Sciences, Beijing, China; 4 Department of Neurosciences, University of California at San Diego School of Medicine, La Jolla, CA, USA

## Abstract

**Background:**

Accumulation of aberrant proteins to form Lewy bodies (LBs) is a hallmark of Parkinson's disease (PD). Ubiquitination-mediated degradation of aberrant, misfolded proteins is critical for maintaining normal cell function. Emerging evidence suggests that oxidative/nitrosative stress compromises the precisely-regulated network of ubiquitination in PD, particularly affecting parkin E3 ligase activity, and contributes to the accumulation of toxic proteins and neuronal cell death.

**Results:**

To gain insight into the mechanism whereby cell stress alters parkin-mediated ubiquitination and LB formation, we investigated the effect of oxidative stress. We found significant increases in oxidation (sulfonation) and subsequent aggregation of parkin in SH-SY5Y cells exposed to the mitochondrial complex I inhibitor 1-methyl-4-phenlypyridinium (MPP^**+**^), representing an *in vitro *cell-based PD model. Exposure of these cells to direct oxidation via pathological doses of H_2_O_2 _induced a vicious cycle of increased followed by decreased parkin E3 ligase activity, similar to that previously reported following S-nitrosylation of parkin. Pre-incubation with catalase attenuated H_2_O_2 _accumulation, parkin sulfonation, and parkin aggregation. Mass spectrometry (MS) analysis revealed that H_2_O_2 _reacted with specific cysteine residues of parkin, resulting in sulfination/sulfonation in regions of the protein similar to those affected by parkin mutations in hereditary forms of PD. Immunohistochemistry or gel electrophoresis revealed an increase in aggregated parkin in rats and primates exposed to mitochondrial complex I inhibitors, as well as in postmortem human brain from patients with PD with LBs.

**Conclusion:**

These findings show that oxidative stress alters parkin E3 ligase activity, leading to dysfunction of the ubiquitin-proteasome system and potentially contributing to LB formation.

## Background

Parkinson's disease (PD) is the most common neurodegenerative movement disorder, affecting approximately 1% of the population over age 60 [1,2]. Histopathology of PD brains shows a progressive loss of dopaminergic (DA) neurons in the substantia nigra and the formation of cytoplasmic inclusions known as Lewy bodies (LBs) and Lewy neurites (LN) [3]. LBs/LNs contain a number of poly-ubiquitin-aggregated proteins, including α-synuclein and parkin, an E3 ubiquitin ligase [4-6]. These alterations are associated with loss of dopaminergic neurons and resulting motor impairment. Interestingly, rare, hereditary mutations can simulate the same phenotype found in patients with sporadic parkinsonism. Recent identification of mutated genes, including α-synuclein and parkin, that are associated with hereditary forms of PD has shed light on the etiology of the disease [7]. Studies show that many mutations in the parkin gene generally result in loss of function and are associated with autosomal recessive juvenile parkinsonism (ARJP) [8,9]. Nonetheless, PD in the vast majority of cases is viewed as a "sporadic" disorder without known cause, although oxidative/nitrosative stress caused by inhibitors of complex I of the mitochondrial electron transport chain, including pesticides, have recently been implicated [2,10].

Increasing evidence indicates that there may be a link between oxidative/nitrosative stress induced by reactive oxygen/nitrogen species (ROS/RNS) and accumulation of aberrant or misfolded proteins associated with ubiquitin-proteasome system (UPS) dysfunction [11-15]. This cellular process involves tagging molecules targeted for degradation with polyubiquitin chains through a series of reactions carried out by ubiquitin enzymes. Parkin is an E3 ubiquitin ligase that is believed to play an important role in the removal and detoxification of abnormally folded proteins [16]. Parkin has a number of putative substrates, including synphilin-1 and parkin itself. Dysfunction of parkin results in abnormal ubiquitination and accumulation of these substrates, which may contribute to cytoplasmic inclusion formation, impairment in UPS activity, and the demise of DA neurons [17,18].

Emerging evidence suggests that oxidative/nitrosative stress-induced mitochondrial as well as UPS dysfunction play pivotal roles in the etiology of many degenerative disorders [13,19,20]. Recent reports from our laboratory and others demonstrated that parkin is S-nitrosylated by excessive nitric oxide (NO) both *in vitro *and *in vivo *[21,22]. This reaction transfers an NO group to critical cysteine thiol(s) to regulate parkin's E3 ubiquitin ligase activity, trigger aberrant protein accumulation, and contribute to neuronal death in PD. Here, we extend these findings to oxidative attack on parkin leading to sulfination (-SO_2_H)/sulfonation (-SO_3_H) reactions.

## Results

### Protein sulfonation recognized *in vitro *by an antibody raised against per-sulfonyl-BSA

**Figure 1:**
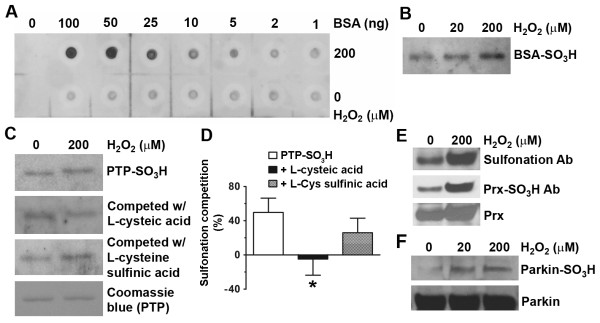
**H**_**2**_**O**_**2**_**-induced cysteine protein sulfonation observed *in vitro***. (A) Dot blot analysis of anti-sulfonation antibody to show dose-response to BSA sulfonation. (B and C) Immunoblot detection with anti-sulfonation pAb of sulfonated purified BSA or PTP1B after oxidative challenge with H_2_O_2_. BSA or PTP1B (10 μM) was exposed to 20 or 200 μM H_2_O_2 _for 30 min. For the competition assay, the anti-sulfonation pAb was incubated with 1 mM cysteic acid or cysteine sulfinic acid at RT for 2 hours prior to the incubation with the membrane. (D) Quantification of the competition assays for PTP1B sulfonation. Sulfonation competition was analyzed by normalizing to the intensity of PTP1B sulfonation (PTP- SO_3_H) without H_2_O_2 _exposure. Data are expressed as mean ± SEM, *n *= 3; *p < 0.05 vs. PTP- SO_3_H by post-hoc ANOVA. (E) Detection of sulfonated Prx from cells under oxidative stress induced by H_2_O_2_. Neuroblastoma SH-SY5Y cells were exposed to H_2_O_2_, followed by immunoblotting with antibodies against per-sulfonyl-BSA, Prx-SO_3 _and Prx, respectively. Both anti-sulfonation antibodies detected a similar level of sulfonated Prx (Prx-SO_3_H). (F) Parkin sulfonation after oxidative stress *in vitro*. Recombinant parkin was exposed to 20 or 200 μM H_2_O_2 _for 30 min, resulting in sulfonation by immunoblot analysis.

To investigate protein posttranslational modifications by oxidation, we generated a broad-spectrum polyclonal antibody (pAb) that could identify protein sulfonation by recognizing epitopes containing cysteine sulfonic acid [Cys(SO_3_^-^)]-modified residues (see Methods). Due to the extremely small size of the specificity-determining side chain of sulfonated cysteine (i.e., methyl sulfonate), a large amount of antigen containing multiple potential epitopes was deemed necessary to boost immunoreactivity for antibody generation. Potential epitopes of interest included the Cys(SO_3_^-^) side chains together with the adjacent peptide bonds, or pairs of adjacent sulfonated cysteines. Our strategy was to oxidize the 35 cysteine residues in BSA to generate per-sulfonyl-BSA, containing up to 35 peptide Cys(SO_3_^-^) groups in various protein sequence environments. The synthesis of per-sulfonyl-BSA was performed following a protocol described previously [23]. Using the antibody thus generated, we initially examined putative sulfonation of a number of cysteine-containing proteins after oxidative stress engendered by H_2_O_2 _*in vitro*. Dot blots with various amounts of BSA were used to examine the dose-dependence of oxidized proteins recognized by the sulfonation pAb (Figure 1A). Next, immunoblot analysis with the sulfonation pAb revealed that an increase in BSA sulfonation correlated to the concentration of H_2_O_2 _that BSA was exposed to (Figure 1B).

### Validation of the sulfonation antibody by *in vitro *competition assays

To further assess the specificity of the sulfonation antibody *in vitro*, we examined another known cysteine-containing protein, protein-tyrosine phosphatase 1B (PTP1B), which has cysteine residues that are known to be oxidized [24]. We used H_2_O_2 _to induce sulfonation of PTP1B and then conducted a competition assay to test for the ability of structurally related amino acids to compete with the cysteic acid epitopes on PTP1B for binding to the anti-sulfonation pAb. We found that there was an approximately 50% increase in PTP sulfonation (PTP-SO_3_H) after exposure to 200 μM H_2_O_2 _*in vitro *(Figure 1C and 1D). Free l-cysteic acid (Cys-SO_3_H) competed virtually completely with PTP-SO_3_H for antibody binding. In contrast, a structurally-related amino acid with negative charges, l-cysteine sulfinic acid (Cys-SO_2_H), competed less well for anti-sulfonation pAb binding. Taken together, these results suggest that the sulfonation antibody was able to recognize oxidant-induced protein sulfonation in a variety of peptide environments, potentially representing a "pan-sulfonation antibody".

### ROS-induced parkin sulfonation observed *in vitro *and *in vivo*

**Figure 2:**
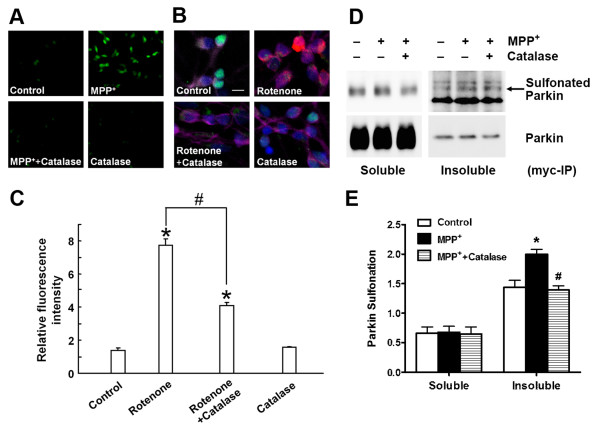
**Oxidative stress-induced parkin sulfonation observed *in vivo***. (A) MPP^+^-induced ROS generation detected by the fluorogenic probe DCF in parkin-overexpressing SH-SY5Y cells. Catalase attenuated ROS production in cells exposed to MPP^+^. (B) ROS production induced by rotenone in a cell-based model of PD in primary striatal neurons. After exposure to 100 nM rotenone for 4 hours, 1 μM HEt was added for 30 min to assess ROS. Then the cells were fixed and immunostained for specific neuronal markers (both MAP2 and NeuN, purple), dopaminergic cells (TH, green), and nuclear DNA (Hoechst, blue). Scale bar, 10 μm. (C) Quantification of ROS intensity by deconvolution microscopy. Rotenone exposure led to ROS production and administration of catalase prior to rotenone reduced ROS generation. Data are expressed as mean ± SEM, *n *= 3; *p < 0.05 against Control; ^#^*p *< 0.05 for Rotenone vs. Rotenone + Catalase by post-hoc ANOVA. (D) Parkin sulfonation in SH-SY5Y cells exposed to MPP^+^. Cell lysates were subjected to immunoprecipitation with anti-*myc *antibody (*myc*-IP). Exposure of parkin-overexpressing SH-SY5Y cells to 200 μM MPP^+ ^for 18 hours resulted in a significant increase in parkin sulfonation in the "Insoluble" fraction. Administration of catalase 1 hour prior to MPP^+ ^exposure prevented parkin sulfonation. Additional bands when probing for sulfonated parkin appeared only in the "Insoluble" fraction. (E) Parkin sulfonation was quantified by normalizing the intensity of sulfonated parkin to total parkin. Data are expressed as mean ± SEM, *n *= 4; *p < 0.01 for MPP^+ ^vs. Control; ^#^*p *< 0.01 for MPP^+ ^vs. MPP^+ ^+ Catalase by post-hoc ANOVA.

To investigate parkin oxidation, we used the sulfonation pAb that we had generated to initially examine parkin sulfonation *in vitro *after oxidative stress engendered by H_2_O_2_. To further ensure the specificity of the antibody in identifying sulfonated proteins, we employed another well-studied antibody, which recognizes solely sulfonated peroxiredoxin (Prx-SO_3_H) [14], as a positive control (Figure 1E). Immunoblot analysis revealed that H_2_O_2 _increased parkin sulfonation *in vitro *in a dose-dependent manner (Figure 1F). Taken together, these results suggest that our "pan-sulfonation antibody" may recognize various sulfonated proteins because it was able to react with proteins containing multiple amino-acid sequences surrounding the sulfonated cysteine residue. To test for the presence and effect of sulfonated parkin in cell-based models of PD, we used either parkin-overexpressing SH-SY5Y cells exposed to toxic levels of the mitochondrial complex I inhibitor--MPP^**+**^, or primary cultures of striatal neurons exposed to the pesticide rotenone. We detected ROS generation in these models with two fluorogenic probes, the cell-permeable fluorogenic dye chloromethyl-2',7' dicholor-dihydrofluorescein diacetate (CM-H_2_DCFDA, DCF) or hydroethidine (HEt; Invitrogen, San Diego, CA). Both cell-based models displayed significant increases in ROS, while pretreatment with cell-permeable catalase, a specific reductase of H_2_O_2_, reduced ROS production and protected dopaminergic neurons from death (Figure 2A-2C and Additional file 1, Figure S1A). We also quantified the increase in DCF fluorescence intensity following MPP^+ ^exposure using a Synergy 4 microplate reader (BioTek, Winooski, VT) and obtained results (Additional file 1, Figure S1B) similar to those previously observed under fluorescence microscopy in a report linking ROS generation to MPP^+ ^exposure [25]. Next, parkin-overexpressing SH-SY5Y cells exposed to MPP^**+ **^were lysed into "Soluble" and "Insoluble" fractions. We enriched for recombinant *myc*-parkin with anti-*myc *antibody, and found evidence that exposure to MPP^+ ^resulted in a significant increase in parkin sulfonation in the "Insoluble" fraction. Pretreatment 1 hour prior to MPP^+ ^exposure with cell-permeable catalase attenuated sulfonation and aggregation of parkin into the insoluble fraction (Figure 2D and 2E). These findings suggest that oxidative stress induced by MPP^+ ^results in parkin sulfonation and aggregation.

### MS analysis of parkin oxidation

It is most important to link structure with function, that is, to identify the critical residues in parkin whose oxidative state regulates the E3 ligase activity. Previous studies of parkin function utilized a predominantly genetic approach and focused on various point mutations of parkin found in familial PD patients [17,26-28]. Such a "bottom-up" approach limits the study to known mutations, and mutations of some of the cysteine-containing enzymes were more arbitrary and lacked genetic support. Therefore, we took an unbiased "top-down" approach by using liquid chromatography MS (LC/MS)-based comprehensive analysis to determine post-translational modifications of cysteine residues in parkin exposed to ROS.

Full-length recombinant human parkin was exposed to various concentrations of H_2_O_2 _*in vitro*, followed by in-solution and in-gel trypsin digestion. The resulting tryptically-digested peptides were subjected to two complementary LC/MS platforms - high resolution nanoLC quadrupole time-of-flight (Q-TOF) MS and high throughput nanoLC ion trap tandem MS (see MS workflow in Additional file 1, Figure S2), as we have described previously [19,21,29]. LC/MS data were then converted into a DeCyder™ MS-compatible format [30]. DeCyder™ MS allows for quantitative proteomics analysis to compare the retention time and m/z value of each identified peak observed in two different LC/MS runs. The MS data generated from either Q-TOF or ion trap were compared to confirm the similarity of their elution profiles. Differentially sulfinated/sulfonated peptides were detected by comparing sample intensity maps and peaks of interest (*m/z *values), selected as precursor ions for subsequent peptide identification and quantification.

**Figure 3:**
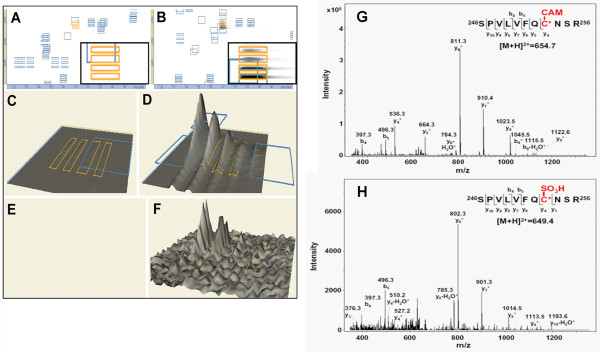
**MS analysis of H**_**2**_**O**_**2**_**-induced parkin sulfonation**. (A) - (F) DeCyder™ MS analysis of high-resolution nanoLC/ESI Q-TOF MS spectra for recombinant GST-parkin. Full length GST-parkin was exposed to H_2_O_2 _(B, D, F and H) and compared to vehicle-treated control (A, C, E and G). MS spectra displayed in DeCyder™ 2D gel format with insets showing higher resolution of MS datasets (A and B), and converted into "3D" format (C and D) representing the alkylated peptide ^246^SPVLVFQCNSR^256^. MS spectral analysis revealed that the sulfonated peptide [^246^SPVLVFQC(SO_3_H)NSR^256^] (F) was not found in the vehicle-treated control (E). Marked blue frames indicate the identified peptides by tandem MS. (G) and (H) Peptide ^246^SPVLVFQCNSR^256 ^identified by high-sensitivity MS/MS ion trap as a double-charged ion with a carbamidomethyl (CAM)-modified cysteine at *m/z *654.7 (G) or sulfonated (SO_3_H) cysteine at *m/z *649.4 (H). Both spectra show characteristic "*y*" and "*b*" series ions. Red asterisks (*) indicates ions that contain the modified cysteine residue.

**Figure 4:**
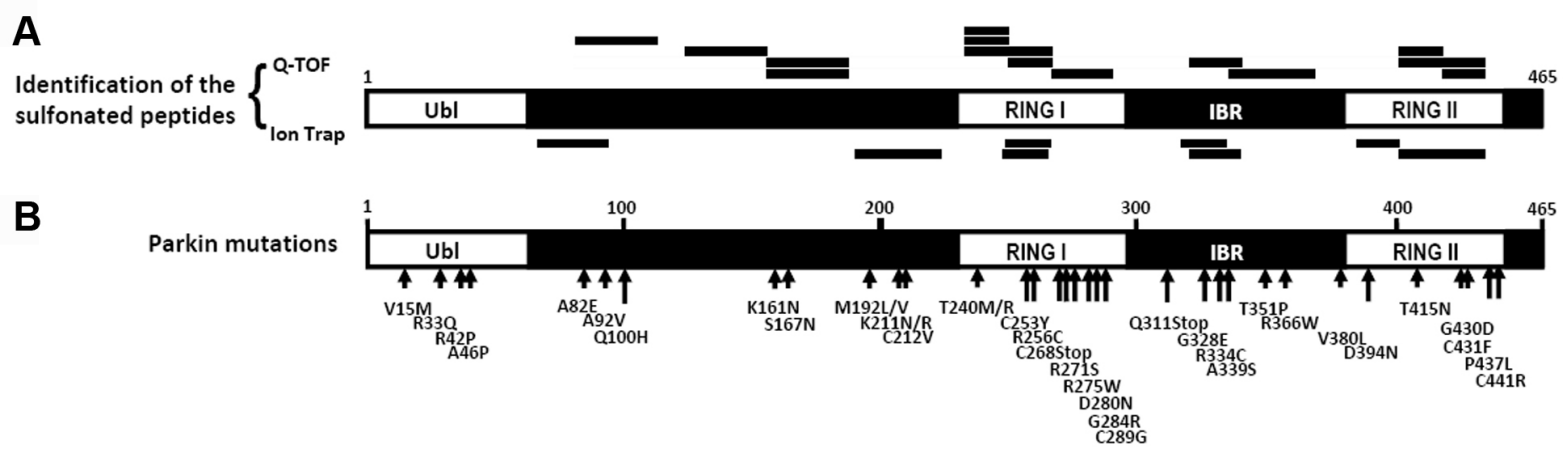
**Mapping locations of parkin oxidation in comparison to parkin mutations found in PD patients**. Parkin (PARK2, UniProt 060260; NCBI AB009973) with 465-aa residues and its various domains indicated [Ubiquitin-like (Ubl), RING I and II, and in-between RING (IBR) domains]. (A) Sulfonated peptides of parkin were identified by either Q-TOF and/or Ion trap MS/MS, and labeled by "─" corresponding to the location of their sequence. Complementary MS analysis demonstrated that the identified cysteine residues in peptide fragments of parkin in the RING and IBR domains (16/22 = 73%) were highly reactive with ROS and formed sulfination/sulfonation derivatives of cysteine. (B), Parkin mutations identified in patients with familial Parkinson's disease (http://www.uniprot.org/uniprot/O60260), revealing high homology with modifications induced by oxidative stress.

We used DeCyder™ MS for multi-spectrum quantitative MS analysis and examined the stoichiometry of the cysteine modifications of parkin based on their chromatography retention time and relative intensity of the identified masses. Q-TOF MS data were displayed in DeCyder™ "2D gel" format with insets showing higher resolution of the parkin RING-I domain peptide, "SPVLVFQCNSR" (Figure 3A-3D). This tryptically-digested parkin peptide was further confirmed by ion trap tandem MS to contain a double-charged ion with a carbamidomethyl (CAM)-modified cysteine (representing alkylation of the free cysteine thiol group by iodioacetamide (IAM) prior to trypsin digestion) at *m/z *654.7 (Figure 3G), or sulfonated (SO_3_H) cysteine at *m/z *649.4 (Figure 3H). The sulfinated/sulfonated cysteine-modified peptides in H_2_O_2_-exposed parkin were compared to the vehicle-treated control and calculated by the DeCyder™ MS PepMatch module. Such quantitative DeCyder™ MS analysis revealed that the sulfonated "SPVLVFQCNSR" peptide (Figure 3F) was not found in the spectra of the vehicle-treated control (Figure 3E). Overall, persistent sulfinated/sulfonated cysteine-containing peptides identified in the H_2_O_2_-exposed parkin by Q-TOF MS and ion trap MS/MS are listed in Table 1 and Table 2, respectively. There 63.6% of the sulfinated/sulfonated cysteine-containing peptides were only found in the H_2_O_2_-exposed parkin. Next, we compared the identified peptide oxidations (sulfination or sulfonation) of parkin with the sites of parkin mutations found in rare familial cases of PD (Figure 4). Almost three quarters (73%) of the sulfinated/sulfonated cysteines in parkin were identified in the RING and IBR (In-Between-RING) domains, indicating that these regions were highly reactive with ROS (Figure 4A). Among these identified cysteines in parkin (shown in Figure 4A), six cysteine residues (C^212^, C^253^, C^268^, C^289^, C^431^, and C^441^) were previously identified from the UniProt archive (http://www.uniprot.org/uniprot/O60260) as bearing point mutations related to rare familial PD (Figure 4B). Mutations of these cysteines alter parkin solubility, intracellular localization, and sensitivity to stress [27,28]. These results are consistent with the notion that the RING and IBR domains contain regions undergoing cysteine modification during oxidative stress that modulate parkin E3 ligase activity and affect ubiquitination-mediated protein degradation, thus contributing to parkin aggregation. Moreover, these findings suggest that sulfonation of these cysteine residues in the more common sporadic cases of PD may mimic the effect of rare mutations observed in familial PD.

**Table 1:** DeCyder MS analysis for Q-TOF MS of recombinant parkin exposed to H_2_O_2_

Domain (Residue #)	Peptide Sequence	**Monoiostopioc *M***_***r ***_**Calculated Observed**		Modifications	Ratio
**RING I**	^235^NITCITCTDVR^245^	1326.59	1326.56	Cys-CAM (C); Cys-SO_2_H (C)	13.2
	
**(238 - 293)**	^235^NITCITCTDVR^245^	1333.55	1333.62	2 Cys-SO_3_H	n/a
	
	^235^NITCITCTDVRSPVLVFQCNSR^256^	2605.19	2605.18†	Cys-CAM; Cys-SO_2_H; Cys-SO_3_H	n/a
	
	^246^SPVLVFQCNSR^256^	1296.61	1296.58	Cys-SO_3_H	n/a
	
	^257^HVICLDCFHLYCVTRLNDR^275^	2367.09	2367.19*	Cys-SO_3_H	3.6

**IBR**	^350^VTCEGGNGLGCGFAFCRECK^369^	2154.86	2154.90	Cys-CAM; Cys-SO_3_H	n/a
	
**(294 - 417)**	^370^EAYHEGECSAVFEASGTTTQAYR^392^	2554.06	2554.09	Cys-SO_3_H	n/a

**RING II**	^428^NGGCMHMK^435^	956.31	956.33	2 Oxidation (Met); Cys-SO_3_H	n/a
	
**(418 - 449)**	^428^NGGCMHMKCPQPQCR^442^	1882.70	1882.77	2 Cys-CAM; 2 Oxidation (Met); Cys-SO_3_H	n/a
	
	^436^CPQPQCR^442^	862.34	862.40	Cys-SO_2_H	n/a

**Others**	^130^DSPPAGSPAGRSIYNSFYVYCK^151^	2410.09	2410.15	Cys-SO_2_H (C)	8.1
	
**(1 - 237)**	^171^QATLTLTQGPSCWDDVLIPNR^191^	2375.14	2375.15	Cys-SO_3_H (C)	n/a
	
	^192^MSGECQSPHCPGTSAEFFFK^211^	2236.89	2236.94	Cys-SO_3_H (C)	69.1
	
	^192^MSGECQSPHCPGTSAEFFFK^211^	2309.90	2309.93	Cys-CAM (C); Oxidation (Met); Cys-SO_3_H (C)	n/a

**Notes**: GST-parkin was exposed to 10 μM (unmarked), 50 μM (*), or 200 μM H_2_O_2 _(†). To compensate for intensity differences between elution profiles of individual experiments, normalization was performed using the measured intensity distribution of the entire peptide population. Our development of this algorithm represents a novel function of DeCyder MS v2.0. The ratio of peptide modification was calculated as the intensity of H_2_O_2_-exposed parkin compared to vehicle-treated control. Peptide sequences in these experiments are determined by mass fingerprint. Legend: n/a, not applicable (since peptide modifications were only identified for the H_2_O_2_-exposed samples); IBR, in-between RING domain; CAM, Carbamidomethyl; Cys, cysteine; Met, methionine.

**Table 2:** DeCyder MS analysis for ion trap MS/MS of recombinant parkin exposed to H_2_O_2_

Domain (Residue #)	Peptide Sequence	Observed (m/z)	**Monoiostopioc *M***_***r ***_**Calculated Observed**		Ratio
**RING I**	^246^SPVLVFQC(SO_2_H)NSR^256^	640.91^2+^	1280.62	1279.81	n/a
	
**(238 - 293)**	^246^SPVLVFQC(SO_3_H)NSR^256^	649.42^2+^	1296.61	1296.83	n/a

**IBR**	^349^KVTCEGGNGLGC(SO_3_H)GFAFCR^366^	623.31^3+^	1865.79	1866.91	3.53
	
**(294 - 417)**	^350^VTC(SO_2_H)EGGNGLGC(SO_3_H)GFAFCREC(SO_2_H)K^369^	721.51^3+^	2161.82	2161.50	n/a

**RING II**	^421^C(SO_3_H)HVPVEK^427^	421.93^2+^	842.40	841.84	13.15
	
**(418 - 449)**	^428^NGGC(SO_2_H)MHM(Oxidation)KCPQPQC(CAM)R^442^	897.89^2+^	1793.69	1793.77	n/a

**Others**	^90^NAAGGC(SO_3_H)EREPQSLTR^104^	819.02^2+^	1635.73	1636.03	5.20
	
**(1 - 237)**	^212^C(SO_3_H)GAHPTSDKETPVALHLIATNSR^234^	822.40^3+^	2465.20	2464.17	1.65

### Oxidative stress induces parkin auto-ubiquitination and aggregation in cell-based and *in vivo *animal models of PD

**Figure 5:**
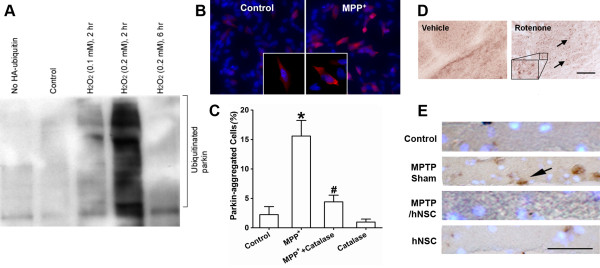
**Oxidative stress-induced parkin auto-ubiquitination and aggregation in PD cell-based and animal models**. (A) Auto-ubiquitination of parkin examined in parkin-overexpressing SH-SY5Y cells exposed to H_2_O_2_. H_2_O_2 _induced dose-dependent and biphasic effects on parkin activity with an initial increase in activity at 2 hours followed by a decrease in activity by 6 hours. (B) and (C) H_2_O_2 _involvement in MPP^+^-induced parkin aggregation in parkin-overexpressing SH-SY5Y cells. Cells plated onto cover slips in 24-well culture plates were immunostained with anti-*myc *mAb for parkin followed by second antibody conjugated to Cy3 fluorescence dye. Cells were then counterstained for nuclear DNA with Hoechst dye 33342. Inset: Higher magnification of cells showing parkin immunoreactive aggregates after MPP^+ ^exposure. Data are expressed as mean ± SEM; *n *= 3, *p < 0.01 for MPP^+ ^vs. Control; ^#^*p *< 0.01 for MPP^+ ^vs. MPP^+ ^+ Catalase by post-hoc ANOVA. Catalase protected parkin from aggregation in parkin-overexpressing SH-SY5Y cells exposed to MPP^+^, indicating that H_2_O_2_-mediated parkin sulfonation may play a role in formation of parkin aggregates. (D) Increased parkin aggregation (arrows) in striatum of rats exposed to rotenone. Inset: High-power view showing parkin immunoreactive aggregates. (E) Increased parkin aggregation in monkeys exposed to MPTP. hNSCs transplanted into MPTP-lesioned monkeys appeared to diminish parkin aggregation (arrow) in the host nigrostriatal system including substantia nigra, similar to that of nonlesioned monkeys with or without hNSCs. Scale bar: D, 100 μm; E, 50 μm.

We next examined whether the ubiquitin E3 ligase activity of parkin was affected by oxidative stress/ROS. We found that exposure of parkin-overexpressing SH-SY5Y cells to H_2_O_2 _resulted in increased auto-ubiquitination within 2 hours, followed by a decrease in activity 6 hours later, reflecting a biphasic effect (Figure 5A). Previously, we reported a similar biphasic pattern in parkin activity after exposure to NO [21]. One possible explanation for this biphasic action is that the free radical modification of parkin initially activates its E3 ubiquitin ligase activity, and the resulting auto-ubiquitination subsequently leads to its inactivation. Excessive ubiquitinated proteins can potentially overwhelm the proteasome and result in aberrant protein accumulation, as has been observed with parkin in LBs of PD patients [4,15].

Emerging genetic studies indicate that a large number of familial PD-linked point mutations of parkin alter its solubility in cells [11,17,26-28]. We thus investigated if the decrease in parkin solubility that we observed in sporadic cases of PD resulted in part from oxidative stress-induced perturbations in the redox state of parkin, producing sulfonation. We studied whether such oxidative stress could affect the cellular localization of parkin in both cell-based and animal PD model systems. We found that parkin-overexpressing SH-SY5Y cells exposed to MPP^+ ^exhibited a dramatic increase in parkin aggregation (Figure 5B and 5C). Administration of catalase prior to MPP^+ ^exposure prevented the aggregation of parkin, consistent with the notion that H_2_O_2_-mediated sulfonation of parkin affects its aggregation state. To confirm that the MPP^+^-induced parkin sulfonation (as shown in Figure 2D and 2E) resulted in parkin aggregation via oxidative stress, we exposed parkin-overexpressing SH-SY5Y cells to various concentrations of H_2_O_2_. Proteins were extracted and separated into "Soluble" and "Insoluble" fractions. These fractions were then subjected to SDS-PAGE and immunoblot analysis to detect parkin. We found a significant increase in parkin in the "Insoluble" fraction and a decrease in the "Soluble" fraction in H_2_O_2_-treated SH-SY5Y cells compared to controls; the increase in insoluble parkin occurred in a dose-dependent manner with regard to hydrogen peroxide exposure (Additional file 1, Figure S3).

To recapitulate the effects of exposure of neurotoxins *in vivo*, we injected 1 mg/kg/day of rotenone intraperitoneally (ip) into rats for 5 days. We found an increase in inclusion body-like parkin immunoreactivity in the striatum of rotenone-exposed rats compared to vehicle-treated controls (Figure 5D). We observed a similar pattern of parkin immunoreactivity in aggregates from monkeys exposed to 1-methyl-4-phenyl-1,2,3,6-tetrahydropyridine (MPTP) (Figure 5E), consistent with our prior findings for α-synuclein aggregates in the nigrostriatal system [31]. Moreover, human neural stem cells (hNSCs) transplanted into MPTP-lesioned monkeys appeared to diminish parkin aggregation in the host substantia nigra, similar to that of nonlesioned monkeys with or without hNSCs (Figure 5E).

### Selective increase in insoluble parkin levels and sulfonation in idiopathic PD brains

**Figure 6:**
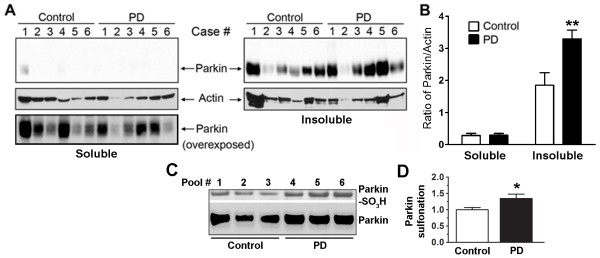
**Selective increase in insoluble parkin levels and sulfonation in idiopathic PD brains**. (A) The "Soluble" and "Insoluble" fractions of brain tissue lysates from the caudate nucleus were blotted for parkin immunoreactivity with PRK8 monoclonal antibody. Immunoblotting revealed that the levels of parkin were significantly increased in the caudate of sporadic PD patients compared to controls. (B) Relative amount of parkin normalized to actin. Data are expressed as mean ± SEM, *n *= 6; **p < 0.01 by post-hoc ANOVA. (C) The "Insoluble" fraction of tissue lysates from postmortem human brains of either normal control subjects or sporadic PD cases were immunoblotted for parkin sulfonation. Pooled brain tissue lysates were obtained from 12 patient samples (see subject information in Additional file 1, Table S2). Increased parkin sulfonation was observed in PD compared to Control. (D) Quantification of parkin sulfonation by normalizing the intensity of sulfonated parkin to total parkin. Data are expressed as mean ± SEM, *n *= 3; *p < 0.05 by post-hoc ANOVA.

Previously, we and others demonstrated that nitrosative stress induced by RNS affects parkin E3 ubiquitin ligase activity [21,22]. This alteration in E3 ligase activity may contribute to LB formation with the accumulation of insoluble, aberrant proteins including parkin and its substrates [4,15,32]. In the present study, since we found that oxidative stress induced by ROS also affects parkin E3 ligase activity, we asked if such insult might also affect aberrant parkin accumulation in idiopathic PD. We initially examined brain tissues from human patients with LB dementia compared to control brains from patients dying of non-CNS causes (Additional file 1, Table S1). Brain tissue was extracted and separated into "Soluble" and "Insoluble" fractions. These fractions were then subjected to SDS-PAGE and immunoblot analysis to detect parkin. We found a significant increase in parkin in the insoluble compared to the soluble fraction of brains obtained from patients with PD and diffuse LBs (Figure 6A and 6B). Note that in PD compared to control brains, the absolute level of parkin increased in the insoluble fraction representing an increase in aggregated parkin.

Next, we examined human postmortem brain tissue for immunodetection of parkin sulfonation in order to demonstrate pathophysiological relevance of increased sulfonation to parkin insolubility in PD. To obtain sufficient tissue for the analysis, we pooled tissue lysates from two cases in each lane on SDS-PAGE for analysis of parkin sulfonation via immunoblot (Additional file 1, Table S2). We found that parkin sulfonation in the "Insoluble" fractions was significantly increased in the PD brains compared to the controls (Figure 6C and 6D). These results demonstrate a significant correlation between increased sulfonation and insoluble parkin in PD brains.

## Discussion

Epidemiological studies indicate that parkinsonism is the most prevalent movement disorder, manifesting multiple risk factors, and predominantly affecting the aged population. Pathogenesis is strongly associated with both genetic susceptibility and environmental factors. Current etiologic hypotheses concerning 'idiopathic' PD favor genetic susceptibility-by-environment (GxE) interactions [7,10]. In the past decade, genetic studies have shown that relatively rare, inherited mutations cause familial forms of the disease; these studies have also provided important insights into the role of molecular networks in the development of hereditary as well as sporadic PD. Emerging evidence suggests that oxidative/nitrosative stress, possibly due to pesticide exposure [10], may serve as a primary event in PD pathogenesis for the more common sporadic or idiopathic form of PD. Recent studies have suggested that oxidative/nitrosative stress may interfere with normal function of the UPS in PD [5,15,33-35]. However, direct evidence for protein modification by free radicals resulting in DA neurodegeneration is limited [36]. In the present study, our direct detection of sulfonated derivatives and subsequent aggregation of parkin in cell-based PD model, an increase in aggregated parkin in rats and primates exposed to mitochondrial complex I inhibitors and in PD brains, in conjunction with finding that parkin function is regulated by ROS, yields mechanistic insight into the chemical reactions of parkin under oxidative stress and their effect on UPS impairment. Conversely, UPS dysfunction has been suggested to cause oxidative/nitrosative stress [37]. Thus, these two processes may develop into a vicious cycle that contributes to aberrant protein accumulation and neurodegeneration in PD.

Here we utilized an unbiased, "top-down" mass spectrometry approach combined with molecular and cell biology methods to elucidate the chemical nature of the posttranslational modifications (PTMs) of cysteine residues in parkin in response to environmental insults. With this approach, we provide evidence specifically linking these PTMs to parkin function. Initially, we discovered parkin sulfonation *in vitro *in response to exposure to pathological concentrations of H_2_O_2_, as well as in cells exposed to MPP^+^. We documented parkin sulfonation by immunoblot analysis using a newly developed antibody against sulfonated peptides, and found that sulfonated parkin was significantly increased in the "Insoluble" fraction of cells exposed to MPP^+^. Catalase significantly reduced sulfonated parkin to levels approximating normal conditions in control cells.

We documented that environmental insults, including the mitochondrial complex I inhibitors MPP^+^, rotenone, or other pesticides, increase levels of oxidative stress, at least in part, in the form of H_2_O_2_. We further found that pathological levels of H_2_O_2 _produce a vicious cycle of increased and then decreased E3 ligase activity. Under these conditions of oxidative stress, we also observed inclusion body-like aggregates of parkin by immunoreactivity in parkin-overexpressing SH-SY5Y cells. As *in vivo *confirmation of these findings, we observed an increase in the amount of aggregated parkin immunoreactivity in the striatum of rotenone-exposed rats and in the nigrostriatal system of MPTP-lesioned monkeys. hNSCs transplanted into MPTP-lesioned monkeys seem to reduce parkin aggregation in the host substantia nigra. This finding, in parallel with immunohistochemistry in the striatum of rotenone-exposed rats, suggests that hNSC transplants offer an opportunity to ameliorate parkin's ubiquitin ligase function by attenuating parkin aggregation, which would otherwise render the protein dysfunctional. Additionally, we found that parkin solubility was significantly decreased in human postmortem PD brains compared to control brains without CNS pathology. Moreover, we demonstrate that an increase in the level of sulfonated parkin correlates with the insolubility of parkin in human PD brains, suggesting pathophysiological relevance of parkin sulfonation in PD. However, there is a caveat in this finding in that we could not demonstrate direct evidence for parkin sulfonation by immunoprecipitation since none of the parkin antibodies available to us were suitable for immunprecipitation. There are at least three possible reasons preventing us from isolating parkin by immunoprecipitation: (1) We have only limited amounts of postmortem human PD brain to start with, especially from the dopamine fiber-rich regions of the corpus striatum (mainly caudate); (2) The amounts of "Soluble" parkin available for immunoprecipitation are relatively low compared to SDS or urea-dissolved "Insoluble" parkin; and (3) It is often observed that SDS or urea used on insoluble proteins may interfere with the ability of the antibodies to immunoprecipitate. Thus, this type of immunoprecipitation experiment is not feasible with current methods. Nonetheless, our findings on tissue lysates do show a significant correlation between increased sulfonation and insoluble parkin in human PD postmortem brains.

Using complementary MS strategies ─ high-resolution Q-TOF MS and high-throughput ion trap MS/MS ─ we mapped the PTMs of parkin under conditions of oxidative stress and encountered sulfination/sulfonation of specific cysteine residues. We found that the RING and IBR cysteine-rich domains manifested these oxidized modifications. These PTMs modulated parkin E3 ligase activity, affected ubiquitination-mediated protein degradation, and contributed to parkin aggregation. Prior mutagenesis experiments on parkin causing mutation-induced protein misfolding have demonstrated that Cys residues both within and outside of the RING-IBR-RING domain are important in maintaining protein solubility [27,28,38]. The two putative sites of S-nitrosylation on parkin, Cys^241 ^and Cys^260^, which were first reported by our group [21] and others, match the predicted S-nitrosylation consensus motif, making them the most likely candidates for physiological modification. S-nitrosylation may also promote further oxidation reactions such as sulfonation, as observed in the present study.

With regard to related nitrosylation/oxidation reactions, S-nitrosylation of Prx interrupts the normal redox cycle of Prx in detoxifying ROS, and thus results in accumulation of cellular peroxides [14]. Therefore, S-nitrosylation of Prx may also contribute to oxidative stress-induced neuronal cell death in PD. These previously published results from our group suggest that the mechanism of interplay between SNO-Prx and Prx-SO_3_H may be different from that of SNO-parkin and parkin-SO_3_H.

A variety of markers and indices in PD patients and animal models have suggested that derangements in mitochondria complex I activity and consequent oxidative/nitrosative stress are important contributors to sporadic PD [39]. At least three mitochondrial complex I inhibitors, including MPTP, paraquat and rotenone, are capable of simulating many features of sporadic PD and provide valuable models for PD investigation [10,40]. Impaired mitochondrial complex I leads to increased oxidative stress, free radical formation, and reduction in ATP formation, rendering neurons more vulnerable to glutamate-related excitotoxicity. ROS/RNS, such as endogenous H_2_O_2 _and NO, are implicated in the pathogenesis of PD. In fact, similar to ROS, NO can lead to secondary oxidative modification on parkin *in vivo *[21,22], and pathological levels of RNS in combination with ROS can produce synergistic cytotoxic effects by irreversibly S-nitrosylating and then further oxidizing proteins as well as other cellular constituents [41,42]. Increased oxidative stress contributes to a cascade leading to DA neuron degeneration predominantly in the pars compacta of the substantia nigra. The occurrence of oxidative stress in PD is supported by both postmortem analyses and studies demonstrating the capacity of oxidizing toxins to induce nigrostriatal degeneration [43,44]. Previous reports have suggested that ROS/RNS can affect proteasomal function [19,26,33,45,46]. Indeed, recent studies by our laboratory and others have shown that nitrosative stress can modulate parkin E3 ubiquitin ligase activity and subsequently impair UPS function [21,22]. Parkin may form a functional complex with PINK1 and DJ-1 [47], but dysfunctional ubiquitination in the face of oxidative/nitrosative stress results in the loss of the intrinsic neuroprotection mechanism, and thus mimics familial PD in the absence of mutation of one of the genes encoding these proteins [21,22]. Moreover, recent work has shown that PINK1 recruits parkin to the outer membrane of impaired mitochondria, and parkin heralds mitophagy through its ubiquitination of outer mitochondrial membrane proteins [48]. As ROS and RNS are closely related and interact with each other, our present results indicate that oxidative and nitrosative stress act similarly on parkin activity. Substantial evidence supports the notion that high levels of basal oxidative stress exist in the substantia nigra pars compacta in the normal brain and that these levels are increased in PD.

As we report in the present study, oxidative stress can lead to sulfonation of the cysteine residues of parkin, which can affect protein tertiary structure, decrease parkin solubility, and affect parkin E3 ligase activity. These changes may contribute to the etiology of sporadic PD. Parkin possesses 35 cysteine residues, corresponding to a cysteine content of 7.5% (human proteomic average is 2.3% [49]). In addition, at least six different cysteine mutants have been experimentally linked to parkin dysfunction (http://www.uniprot.org/uniprot/O60260). These findings suggest that cysteine residues in parkin are essential for its function, including protein folding and ubiquitination. Yet these cysteines also predispose this highly-expressed CNS protein to chemical modifications under nitrosative and oxidative stress, including S-nitrosylation and sulfonation. We speculate that such oxidative changes may result in structural changes in the protein similar to those produced by hereditary cysteine substitutions linked to parkinsonism, e.g., substitution of cysteine to Y(212), Y(253), stop(256), G(289), F(431), or R(441). In fact, we have found evidence for cysteine residues that are sulfonated in parkin are associated with aggregation of the protein. Hence, oxidation of structurally or functionally critical cysteine residues might represent a molecular point of convergence in the pathogenesis of PD, connecting hereditary mutations that affect parkin solubility and function with adverse environmental insults resulting in similarly detrimental oxidative modifications of parkin at a posttranslational level.

## Conclusions

In summary, we report here that parkin sulfonation produces the appearance of LB-like aggregates during oxidative stress from environmental insults. This study provides mechanistic insight into the action of biologically significant PTMs that regulate protein degradation and contribute to neurodegenerative disorders. As alluded to above, several of the cysteine thiol sulfinated/sulfonated peptides of parkin identified by our MS techniques were previously reported as mutated parkin cysteine residues in familial forms of PD. Mutations of these cysteines alter parkin solubility, intracellular localization, and sensitivity to stress [27,28]. Thus, we suggest that PTMs, including S-nitrosylation and subsequent sulfination/sulfonation [42], may give rise to similar alterations in tertiary structure as rare hereditary mutations, thus providing a mechanistic link between genetic and sporadic forms of PD. Understanding the molecular mechanisms that give rise to these PTMs on parkin holds promise for the development of targeted anti-oxidant therapies for PD.

## Methods

### Expression of recombinant parkin

Recombinant parkin was expressed and purified as previously described [21]. Briefly, full-length parkin cDNA was cloned into a pGEX-4T-2 vector, and the constructs transduced into BL21 cells. Expression of recombinant GST-parkin was induced by isopropyl β-D-1-thiogalactopyranoside (IPTG) according to the manufacturer's instructions (GE Healthcare, Uppsala, Sweden). After cell lysis, GST-parkin was purified on a column of glutathione Sepharose beads and further treated with thrombin to remove GST. Parkin (1 μg) was exposed to 0, 10, or 200 μM H_2_O_2 _at room temperature (RT) for 30 minutes (min) and then immunoblotted to detect parkin sulfonation.

### Generation and evaluation of an anti-sulfonation polyclonal antibody

A polyclonal antibody (pAb) was developed in order to recognize diverse epitopes containing cysteine sulfonic acid [Cys(SO_3_^-^)] derivatives of intact proteins. Unlike other commercial sulfonation antibodies that react with only one oxidized protein (for instance of Prx-SO_3_H), we attempted to design a polyclonal antibody that would react with multiple sulfonated peptides. For this purpose, we reasoned that BSA, which has multiple cysteine residues, could serve as a scaffold for the generation of several sulfonation antigens--per-sulfonyl-BSA. In brief, BSA (200 mg) was suspended in 8.88 ml of highly purified DMSO. Then 1 ml of 10 mg/ml iodine in DMSO was added, followed by 0.12 ml of 36% HCl. The mixture was then incubated at 45°C for 24 hours while shaking gently. After cooling to RT, the product was precipitated with 20 ml of cold acetone at 4°C for 1 hour. The precipitate was washed with 10 ml of acetone twice as above, and subsequently washed twice with cold methanol at 1-hour intervals at 4°C. The last precipitate was dried under a stream of nitrogen.

The final product was analyzed by mass spectrometry to verify the oxidative BSA modifications. The absence of reducible thiols in the product thus obtained was confirmed by Ellman's reagent (DTNB). Sulfonated BSA was used as the antigen and submitted to a commercial vendor, Abgent (San Diego, CA), to generate anti-sulfonation polyclonal anti-serum from rabbits with an ELISA titer higher than 1:10,000.

The anti-sulfonated BSA pAb was purified using protein A/G-agarose. Briefly, rabbit anti-serum containing the anti-sulfonated BSA pAb was mixed with an equal volume of 100 mM phosphate buffer (pH 7.4) and incubated with protein A/G-agarose at 4°C for 2 hours. After washing the beads with 100 mM phosphate buffer, the anti-sulfonated BSA pAb was eluted five times with 100 μl volumes of 100 mM glycine solution (pH 2.8), followed by neutralization of each eluate with 5 μl of 1 M Tris solution (pH 8.3).

Next, we sought to minimize any rabbit anti-serum containing antibodies that recognized the reduced form of BSA, i.e., BSA that had been reduced and converted to its CAM derivative by alkylation with IAM. To deplete any anti-reduced BSA pAb, the eluted anti-serum was incubated with reduced BSA-agarose, prepared as follows: 1 ml NHS (N-hydroxysuccinimide)-activated agarose was incubated with a 2 ml volume of BSA (10 mg/ml) in 100 mM phosphate buffer (pH 7.4) for 1 hour at RT; residual BSA was then removed by centrifugation. Following this step, 2 M ethanolamine (pH 7.4) was added to the beads and incubated at RT for 15 min. After washing the beads with 50 mM Tris (pH 7.4), 50 mM dithiothreitol (DTT) was added for 1 hour at RT, followed by washing with 1 mM DTT. Then 50 mM IAM was added for 1 hour at RT. Finally, the resulting anti-sulfonated BSA pAb was incubated with an equal volume of reduced BSA-agarose overnight at 4°C for further purification. After centrifugation, the supernatant was saved as the anti-sulfonation pAb. Dot blotting was performed to determine the dose-response of the pAb to antigen. To further evaluate the specificity of the anti-sulfonation pAb, competition assays were performed using chemicals with structures similar to the envisaged epitopes containing Cys(SO_3_^-^). Briefly, 10 μM BSA or another cysteine-containing protein, such as recombinant PTP1B in 50 mM Tris (pH 7.4), was exposed to 20 or 200 μM H_2_O_2 _for 30 min at RT. The resulting sulfonated BSA or PTP1B was then gel electrophoresed and transferred to nitrocellulose membrane for immunoblotting. The membrane was incubated with anti-sulfonation pAb at 4°C overnight. For the competition assays, the anti-sulfonation pAb was incubated with 1 mM l-cysteic acid or l-cysteine sulfinic acid at RT for 2 hours prior to immunoblotting.

For cell-based assays to assess the anti-sulfonation pAb, SH-SY5Y cells were exposed to 200 μM H_2_O_2_, and the resulting sulfonated proteins in cell lysates were subjected to electrophoresis for immunoblotting with the pAb. Peroxiredoxin (Prx) and sulfonated Prx (Prx-SO_3_H; Lab Frontier, Seoul, Korea) served as controls [14].

### Cell-based and animal models of PD

(i) PD cell-based models: *Myc*-tagged, parkin-overexpressing SH-SY5Y cells [21] were grown in Dulbecco's modification of Eagle's Medium (DMEM) containing 10% fetal bovine serum (FBS) and 500 μg/ml geneticin in a 5% CO_2 _atmosphere. At 70% confluence, the medium was removed and cells were incubated with the mitochondrial complex I inhibitor, MPP^**+ **^(Sigma, St. Louis, MO), in FBS-free DMEM for 18 hours. Primary cultures of the enriched striatal neurons were prepared and maintained for 14 days *in vitro *(DIV), as previously described [50] with minor modifications. Briefly, to prepare primary cultures, striatal tissues were dissected out from E16-18 time-pregnant Sprague-Dawley rats and dissociated in 0.05% trypsin for 35 min at 37°C. Striatal neurons were seeded in DMEM supplemented with heat-inactivated FBS and Ham's F12 (ratio 8:1:1), 24 U/ml penicillin, 24 μg/ml streptomycin, and 24 mM HEPES, pH 7.4 at a density of 0.67 × 10^6 ^cells per 35-mm dish containing poly-l-lysine (PLL, Sigma, St. Louis, MO) coated glass cover slips. The cultures were replaced the following day with DMEM supplemented with 1% B27 neurobasal medium and 0.5 mM glutamate, and incubated in a 5% CO_2_, 95% air humidified atmosphere at 37°C. Striatal neurons were exposed to 100 nM rotenone for 4 hours. To prevent H_2_O_2 _accumulation, 100 U/ml catalase (Sigma, St. Louis, MO) was added to the medium 1 hour prior to MPP^**+ **^or rotenone exposure.

(ii) PD animal models: All animal experiments were performed as described previously [21] according to guidelines set by the relevant institutional Animal Care and Use Committees of the collaborating institutions. Briefly, Sprague-Dawley rats weighing 200-250 g were divided into two groups (*n *= 10 per group) to receive ip injections of freshly prepared rotenone (1 mg/kg/day in 50% DMSO and 50% polyethylene glycol) or vehicle only (as the control group) daily for 5 days. MPTP-lesioned monkey brain slices were generously provided by Dr. Evan Y. Snyder of the Sanford-Burnham Medical Research Institute with details of the preparation previously described [31].

### Immunoblotting parkin from fractionated cells and human brains

Autopsied human brain samples were analyzed following the guidelines of the relevant Institutional Review Boards. Cell lysates and PD brain tissue homogenates were sequentially fractionated as previously described [28,51] with minor modifications. *Myc*-tagged, parkin-overexpressing stable SH-SY5Y cells were lysed with 1% Triton X-100 in phosphate buffered saline (PBS) containing a cocktail of protease inhibitors (Sigma, St. Louis, MO) and sedimented at 22,000 g at 4°C for 15 min. Supernatant from the initial fractionation was harvested as the "Soluble" fraction, and the remaining pellet was washed once with 1% Triton X-100 in PBS before re-extraction with 1% SDS in PBS. After sedimentation at 22,000 g for 15 min at RT, the supernatant was collected as the "Insoluble" fraction. Equal amounts of protein from the "Soluble" and "Insoluble" fractions were used for immunoprecipitation (IP) with anti-*myc *mAb-Agarose (Santa Cruz, CA) in order to detect parkin. For IP, 1 mg of protein from the "Soluble" fraction, or 200 μg of protein from the "Insoluble" fraction diluted 10-fold with 1% Triton X-100 in PBS, was incubated with 40 μl of a 25% slurry of anti-*myc *mAb-agarose overnight at 4°C. After washing with 1% Triton X-100 in PBS on ice, the beads were re-suspended in an aliquot of SDS sample buffer and the eluant collected for detection of parkin sulfonation by electrophoresis under reducing conditions with subsequent immunoblot analysis.

Similarly, brain tissue from PD patients or control subjects without CNS disease was fractionated with 1% Triton X-100 (designated the "Soluble" fraction) followed by 8 M urea (the "Insoluble" fraction) in 0.5 M NaCl and 50 mM Tris at pH 7.4. Equal amounts of protein from the different fractions were loaded for immunoblotting with anti-parkin monoclonal (m)Ab (PRK8, Cell Signaling Tech. Danvers, MA). For all experiments, protein concentration was determined with a BCA protein assay kit.

### ROS detection and immunocytochemistry

Cellular localization of parkin was examined in cultures by immunocytochemistry and confocal microscopy, as previously described [28]. Parkin-overexpressing SH-SY5Y cells were plated onto cover slips in 24-well culture plates. After two days, cells were incubated with 200 μM MPP^**+ **^in FBS-free DMEM for 18 hours or with 100 nM rotenone for 4 hours, with or without the administration of 100 U/ml catalase 1 hour prior to exposure to neurotoxins. For ROS detection, during the final 30 min of exposure to either MPP^+ ^or rotenone, 5 μM cell-permeable fluorogenic dye DCF or 1 μM HEt was applied to the cells, as suggested by the manufacturer. Cells were fixed in 4% paraformaldehyde (PFA), permeabilized with 0.1% Triton X-100, and incubated with anti-*myc *mAb for parkin, NeuN and MAP-2 mAb to identify neurons, or anti-tyrosine hydroxylase (TH) antibody for labeling dopaminergic neurons in primary striatal cultures, followed by second antibody conjugated to various fluorescent dyes. Cells were then counterstained for nuclear DNA with Hoechst dye 33342 (Invitrogen, San Diego, CA).

Immunohistology on tissue sections was performed with standard methods using a primary pAb for parkin (2132, Cell Signaling Tech, Danvers, MA) at a dilution of 1:500 plus a secondary Ab at a dilution of 1:200 with the Vector ABC kit (Burlingame, CA). Monkey brain sections were counterstained with Hoechst 33342. Fluorescent images were acquired on an Olympus X81 spinning-disc confocal microscope equipped with a Hamamatsu deep-cooled monochrome EM camera (C9100-13) and 3I-Slidebook™ software analysis package (Denver, CO). Immunostaining that omitted primary antibody and SH-SY5Y cells transfected with empty vector pcDNA3.1 were included as negative controls. To determine the number of cells containing parkin aggregates, ~200 *myc*-parkin positive cells were counted from three independent cover slips per experiment.

### Polyubiquitination assays

For cell-based ubiquitination assays, parkin-overexpressing SH-SY5Y cells were transfected with hemagglutinin (HA)-tagged ubiquitin, exposed to various concentrations of H_2_O_2_, and then examined at different time points. The proteasome inhibitor MG132 (10 μM) was added to block degradation of poly-ubiquitinated proteins during the assay. Cell lysates were subjected to immunoprecipitation with anti-*myc *mAb for parkin, followed by Western blotting against HA for ubiquitin.

### Mapping PTMs of cysteine residues in parkin

(i) Protein digestion: Recombinant human parkin was exposed to pathological doses of H_2_O_2 _*in vitro *followed by in-solution or in-gel trypsin digestion, as described previously [19,21]. Protein digestion was carried out by in-solution digestion or by DigestPro in-gel digestion using a robotic workstation (Intavis, Koeln, Germany).

(ii) High-resolution mass spectrometry: LC-MS analyses were carried out using a high-resolution (5 ppm) Q-TOF API-US mass spectrometer (Waters Micromass, Manchester, UK) with an on-line nanoLC system (LC Packings, Sunnyvale, CA), as previously described [21]. We used a 75 μm I.D. × 15 cm PepMap C18 analytical column (LC Packings, Sunnyvale, CA) with 3-μm particle size. Mobile phase A was 2% acetonitrile (ACN)/0.1% formic acid (FA), while mobile phase B was 80% ACN/0.1% FA. The gradient was 2% ~ 40% mobile phase B in 75 min, followed by 100% mobile phase B for 5 min. The flow rate was set at 0.2 μl/min. Mass spectra of untreated parkin and H_2_O_2_-exposed parkin were analyzed for PTMs, including cysteine alkylation or sulfination (R-SO_2_H)/sulfonation (R-SO_3_H) and methionine oxidation using an in-house Mascot 2.0 server (Matrix Science Inc., London, UK). The modifications of tryptically-digested peptides of parkin were further confirmed by tandem MS spectra analysis as described below.

(iii) High-throughput ion trap tandem MS: We used packed Picofit capillary column with an integral spray tip (Picofit, 15 μm tip, New Objective, Woburn, MA) for reversed-phase nanoLC MS/MS. The column had an internal diameter of 75 μm, was 15 cm long, and packed with 5 μm C18 AQUASIL. A linear gradient elution was used, from buffer A (0.1% FA in water) to 50% buffer A + 50% buffer B (0.1% FA in ACN) in 100 min, and flow rate was set at 0.2 μl/min. Two ion-trap mass spectrometers were used for maximizing peptide detection: an LTQ linear ion trap mass spectrometer (Thermo Electron, San Jose, CA) and an HCTultra PTM discovery system (Brüker Daltonics, Bremen, Germany). The instruments were operated in a data-dependent mode, in which the first and second strongest ions were sequenced in each cycle with dynamic exclusion enabled and the collision energy set at 35%. For the nanoLC-MS/MS analysis by using an LTQ linear ion trap LC/MS, recombinant proteins were digested with 0.04 μg/μl trypsin for 15 hours. The resulting tryptic digests were then on-line separated on a 5-cm packed PicoFrit capillary column (New Objective, Woburn, MA) with an Eksigent nanoLC system (Dublin, CA). Initially, it was eluted with H_2_O/ACN/FA (98:2:0.1, v/v/v) for 5 min, then a linear gradient increased ACN to 55% by 60 min, and finally decreased ACN to 2% by 65 min. MS/MS spectra were acquired in the data-dependent scanning mode with one full scan followed by four MS/MS scans on the most intense precursor ions with dynamic exclusion enabled. MS/MS data were then further analyzed with SEQUEST Sorcerer software (Sage-N Research, Milpitas, CA).

(iv) DeCyder™ MS analysis: LC/MS data generated by either Q-TOF MS or ion trap MS/MS were converted into a DeCyder™ MS v2.0 (GE Bioscience, Uppsala, Sweden) compatible format, as previously described [30], and the MS profiles were aligned based on the m/z and retention time values to ensure the reproducibility and consistency of the LC spectral profiles. Differentially sulfinated/sulfonated peptide candidates were detected and quantified by comparing the signal intensity maps generated by DeCyder™ MS software, and peaks of interest (*m/z *values and charge states) were selected as precursor ions for subsequent peptide identification.

## Competing interests

The authors declare that they have no competing interests.

## Authors' contributions

Author contributions: ZG and SAL designed the research; ZG, FM, DY, WW, JR, YS, YM and BM performed experiments; ZG, FM, YS, JK, YM, BM, EM and SAL analyzed the data. ZG, FM and SAL wrote the paper. All authors have read and approved the final manuscript.

## Supplementary Material

Additional file 1 **Additional Figures and Tables.Figure S1:** ROS production in cellular PD models. (A) ROS production in primary rat striatal neurons induced by rotenone. After exposure to 100 nM rotenone for 4 hours, 1 μM hydroethidine (HEt) was added for 30 min to assess ROS (red). Then the cells were fixed and immunostained for specific neuronal markers (MAP2 and NeuN, purple), dopaminergic cells (TH, green), and nuclear DNA (Hoechst, blue). Scale bar, 80 μm. (B) Quantification of ROS generation in SH-SY5Y cells by fluorescence microplate reader. MPP^+ ^exposure led to ROS production. Administration of catalase prior to MPP^+ ^exposure reduced ROS generation Values are expressed as mean ± SEM, *n *= 3; *p < 0.05 against Control; ^#^*p *< 0.05 for MPP^+ ^vs. MPP^+ ^+ Catalase by post-hoc ANOVA.
**Figure S2:** Mass spectrometry (MS) workflow to identify parkin modifications. GST-parkin was exposed to H_2_O_2 _*in vitro*, followed by trypsin digestion. The digested peptides were subjected to on-line nanoLC attached to either high-resolution Q-TOF MS or high sensitivity ion trap tandem MS analysis. These LC/MS data were then converted into a DeCyder™ MS-compatible format for proteomic comparison (the *m/z *value of each identified peak was compared between LC/MS runs).
**Figure S3:** Decreased parkin solubility in SH-SY5Y cells. *Myc*-parkin-overexpressing SH-SY5Y cells were exposed to 0, 0.2 or 1 mM H_2_O_2 _for 1 hour. Cell lysates were separated into "Soluble" and "Insoluble" fractions, followed by Western blotting against *myc *to identify parkin. After exposure to H_2_O_2_, the solubility of *myc*-parkin decreased dramatically in SH-SY5Y cells. Coomassie blue staining of the gels was used to ensure equal protein loading.
**Table S1:** List of human brain subjects for parkin immunoblotting analysis.
**Table S2:** List of human brain subjects for immunoblotting analysis of parkin sulfonation Click here for file
