# Supplementary material for: Oxidation of the cysteine-rich regions of parkin perturbs its E3 ligase activity and contributes to protein aggregation
Source: Mol Neurodegener. 2011 May 19;6:34. doi: 10.1186/1750-1326-6-34 (PMC3120712; doi:10.1186/1750-1326-6-34)
Supplement: Additional file 1 — Additional Figures and Tables.Figure S1: ROS production in cellular PD models. (A) ROS production in primary rat striatal neurons induced by rotenone. After exposure to 100 nM rotenone for 4 hours, 1 μM hydroethidine (HEt) was added for 30 min to assess ROS (red). Then the cells were fixed and immunostained for specific neuronal markers (MAP2 and NeuN, purple), dopaminergic cells (TH, green), and nuclear DNA (Hoechst, blue). Scale bar, 80 μm. (B) Quantification of ROS generation in SH-SY5Y cells by fluorescence microplate reader. MPP+ exposure led to ROS production. Administration of catalase prior to MPP+ exposure reduced ROS generation Values are expressed as mean ± SEM, n = 3; *p < 0.05 against Control; #p < 0.05 for MPP+ vs. MPP+ + Catalase by post-hoc ANOVA. Figure S2: Mass spectrometry (MS) workflow to identify parkin modifications. GST-parkin was exposed to H2O2 in vitro, followed by trypsin digestion. The digested peptides were subjected to on-line nanoLC attached to either high-resolution Q-TOF MS or high sensitivity ion trap tandem MS analysis. These LC/MS data were then converted into a DeCyder™ MS-compatible format for proteomic comparison (the m/z value of each identified peak was compared between LC/MS runs). Figure S3: Decreased parkin solubility in SH-SY5Y cells. Myc-parkin-overexpressing SH-SY5Y cells were exposed to 0, 0.2 or 1 mM H2O2 for 1 hour. Cell lysates were separated into "Soluble" and "Insoluble" fractions, followed by Western blotting against myc to identify parkin. After exposure to H2O2, the solubility of myc-parkin decreased dramatically in SH-SY5Y cells. Coomassie blue staining of the gels was used to ensure equal protein loading. Table S1: List of human brain subjects for parkin immunoblotting analysis. Table S2: List of human brain subjects for immunoblotting analysis of parkin sulfonation [file 1750-1326-6-34-S1.DOC]

**Additional file 1, Figure S1**

**Additional file 1, Figure S2**

**Additional file 1, Figure S3**

**Table S1 List of human brain subjects for parkin immunoblotting analysis**

| Group | Diagnosis | Case # | PMI (hour) | Age (year) | Gender |
| --- | --- | --- | --- | --- | --- |
| Control | Normal | 1 | 2 | 71 | Male |
|  | Normal | 2 | 9 | 102 | Female |
|  | Normal | 3 | N/A | 87 | Male |
|  | Normal | 4 | 12 | 97 | Female |
|  | Normal | 5 | 8 | 63 | Female |
|  | Normal | 6 | 24 | 69 | Male |
| PD | PD, idiopathic; AD-LBV | 1 | 3 | 75 | Female |
|  | PD, idiopathic; Amyloid angiopathy | 2 | 24 | 74 | Male |
|  | PD with diffuse Lewy bodies | 3 | 12 | 73 | Male |
|  | PD, idiopathic | 4 | 5 | 82 | Female |
|  | PD, idiopathic; AD-LBV | 5 | N/A | 89 | Female |
|  | PD, idiopathic | 6 | 8 | N/A | Male |

PMI, postmortem interval; N/A, not available; AD-LBV, Alzheimer’s disease with Lewy body variant.

**Additional file 1, Table S1**

| **Table S2 List of human brain subjects for immunoblotting analysis of parkin sulfonation** | | | | | | | | |
| --- | --- | --- | --- | --- | --- | --- | --- | --- |
| Group | Pool # | Case # | Diagnosis | PMI (hour) | | Age (year) | | Gender |
| Control | 1 | 1 | Normal | | 2 | 71 | Male | |
|  |  | 2 | Normal | | 9 | 102 | Female | |
|  | 2 | 3 | Normal | | N/A | 87 | Male | |
|  |  | 4 | Normal | | 8 | 63 | Female | |
|  | 3 | 5 | Normal | | 72 | 83 | Female | |
|  |  | 6 | Normal (infarct; acute ischemia) | | 12 | 77 | Female | |
| PD | 4 | 7 | PD | | 6 | 77 | N/A | |
|  |  | 8 | PD | | 12 | 85 | N/A | |
|  | 5 | 9 | PD, idiopathic | | 8 | N/A | Male | |
|  |  | 10 | PD, idiopathic; AD | | 3 | 75 | Female | |
|  | 6 | 11 | PD | | 4 | 75 | N/A | |
|  |  | 12 | PD | | 4 | 75 | N/A | |

**Additional file 1, Table S2**
